# Suppression of the toll-like receptor 7-dependent type I interferon production pathway by autophagy resulting from enterovirus 71 and coxsackievirus A16 infections facilitates their replication

**DOI:** 10.1007/s00705-017-3592-x

**Published:** 2017-10-19

**Authors:** Jie Song, Yajie Hu, Jiaqi Li, Huiwen Zheng, Jingjing Wang, Lei Guo, Haijng Shi, Longding Liu

**Affiliations:** 1 Institute of Medical Biology, Chinese Academy of Medical Science and Peking Union Medical College, 935 Jiaoling Road, Kunming, 650118 Yunnan China; 2 Yunnan Key Laboratory of Vaccine Research and Development on Severe Infectious Diseases, Kunming, 650118 China

## Abstract

Toll-like receptors (TLRs) act as molecular sentinels, detecting invading viral pathogens and triggering host innate immune responses, including autophagy. However, many viruses have evolved a series of strategies to manipulate autophagy for their own benefit. Enterovirus 71 (EV71) and coxsackievirus A16 (CA16), as the primary agents causing hand, foot and mouth disease (HFMD), can induce autophagy leading to their replication. Therefore, the objective of this study was to investigate whether enhanced viral replication caused by autophagy in EV71 and CA16 infections was associated with a TLR-related signaling pathway. Our results demonstrate that complete autophagy and incomplete autophagy were observed in human bronchial epithelial (16HBE) cells infected with EV71 and CA16. Moreover, suppression of autophagy by the pharmacological modulator 3-MA significantly and clearly decreased the survival rates and viral replication of EV71 and CA16 in 16HBE cells. Inhibition of autophagy also enhanced the expression of molecules related to the TLR7-dependent type I interferon (IFN-I) production pathway, such as TLR7, MyD88, IRF7 and IFN-α/β. Finally, immunofluorescence staining demonstrated that TLR7 endosome marker M6PR levels were clearly reduced in EV71- and CA16-infected cells, while they were markedly elevated in infected cells treated with 3-MA. These findings suggest that increased EV71 and CA16 replication meditated by autophagy in 16HBE cells might promote degradation of the endosome, leading to suppression of the TLR7-mediated IFN-I signaling pathway.

**Electronic supplementary material:**

The online version of this article (doi:10.1007/s00705-017-3592-x) contains supplementary material, which is available to authorized users.

## Introduction

Enterovirus 71 (EV71) and coxsackievirus A16 (CA16) are the primary pathogens associated with hand, foot and mouth disease (HFMD), which is generally considered to be a common exanthematous illness that is mostly observed in children under 5 years old throughout the world [1]. HFMD is normally asymptomatic is manifested as a clinically mild and self-limiting disease characterized by fever, loss of appetite and vesicular lesions on the palms of the hands, soles of the feet, oral mucosa and tongue [2]. Nevertheless, over the past three decades, the Asia-Pacific region has experienced several widespread and severe outbreaks of HFMD with a high incidence of fatal cardiopulmonary and neurological complications, such as pulmonary edema, cardiac dysfunction, cardiac failure, acute flaccid paralysis, aseptic meningitis, cerebellar ataxia, meningoencephalitis, and encephalomyelitis, which ultimately lead to high numbers of deaths. HFMD is therefore an important public health problem in much of the region [3]. Although both EV71 and CA16 are small non-enveloped RNA viruses that belong to the species *Enterovirus A* of the family *Picornaviridae* and share a high degree of genetic similarity, the clinical manifestations and pathogenesis of HFMD caused by these two viruses differ in some respects [4]. Generally, EV71 infection easily progresses to serious central nervous system (CNS) complications and even leads to death, whereas CA16 infection is often self-limiting and results in mild clinical symptoms [5]. Thus, extensive studies have focused on EV71 because of the conspicuous morbidity and mortality caused by this virus in recent years, whereas CA16 has received less attention. However, epidemiological surveys indicate that over half of the increasing number of lethal HFMD cases and outbreaks in China have been caused by EV71 and CA16 circulating alternately or together [6, 7]. There is also accumulating evidence that CA16 infection can cause severe CNS complications and death [6]. Hence, in-depth investigations of the different pathogenic mechanisms associated with EV71 and CA16 infections are necessary to develop effective vaccines and antiviral therapies against EV71 and CA16.

Autophagy, which literally means “to eat oneself,” is a major biological process that maintains cellular homeostasis, balances cellular metabolism, and promotes cellular survival under stressful conditions by delivering old proteins or damaged organelles for lysosomal degradation and recycling [8]. It is a highly regulated process that initially begins with the engulfment of portions of the cytosol into a characteristic double-membrane vacuole, called a phagophore. The phagophore subsequently causes autophagy-related proteins and damaged organelles to aggregate, followed by nucleation and elongation to form autophagosomes. The autophagosomes then gradually mature and become more acidic, and they then fuse with lysosomes to become autolysosomes. The sequestered contents are degraded inside the autolysosomes by lysosomal hydrolases for recycling [9]. Abnormalities in this process may result in various diseases, such as diabetes, heart disease, cancer, and neuron degeneration [10].

There is increasing evidence that autophagy can act as an immune surveillance mechanism that delivers viral antigens or components to TLR-containing endosomal/lysosomal compartments that are enriched with immune sensors for degradation during infection with viruses, such as herpes simplex virus (HSV) 1, Sindbis virus, and tobacco mosaic virus [11]. However, some viruses, such as poliovirus, Kaposi’s sarcoma-associated herpesvirus (KSHV), Epstein-Barr virus, hepatitis B virus, dengue virus, and human immunodeficiency virus (HIV) 1, have developed strategies to subvert the autophagic machinery for viral replication and to provide a survival advantage in host cells [12]. Moreover, previous studies have indicated that EV71 and CA16 can induce autophagy to promote replication, but the underlying mechanisms of autophagy induced by EV71 and CA16 have still not been elucidated [13, 14]. Viruses can also utilize autophagy to improve replication in host cells by linking to pattern-recognition receptors (PRRs), including Toll-like receptors (TLRs) [15]. For example, HSV-1-mediated autophagy sustains replication by early activation of the TLR2-MyD88 signaling pathway [16]. Therefore, we hypothesized that autophagy caused by EV71 and CA16 infections is related to the TLR signaling pathway, which might trigger differences in immune responses.

## Materials and methods

### Cell culture and virus

Human bronchial epithelial cells (16HBE) were purchased from Jennino Biological Technology (Guangzhou, China) and cultured at 37 °C in a humidified atmosphere of 95% air and 5% CO_2_ in Dulbecco’s modified Eagle medium (DMEM; Corning, USA) containing 10% fetal bovine serum (FBS; Gibco, USA) and 100 U of penicillin and 100 μg of streptomycin per ml. After sequential passages of 16HBE cells using a 0.25% trypsin/EDTA solution (Invitrogen, USA), approximately 1 × 10^5^ cells per well were seeded into 6-well culture plates and grown to 80% confluence. The cells were then infected at a multiplicity of infection (MOI) of 1 with EV71 (sub-genotype C4, GenBank: EU812515.1) or the CA16-G20 strain (sub-genotype B, GenBank: JN590244.1), which were isolated from an epidemic in Fuyang, China, in 2008 and from an HFMD patient in Guangxi, China, in 2010, respectively. In addition, 16HBE cells were also treated with 3-methyladenine (3-MA, an autophagy inhibitor; Sigma, USA), rapamycin (an autophagy activator; Sigma, USA) or NH_4_Cl (an autophagy inhibitor; Sigma, USA). Cells were harvested at 0, 6, 12 and 24 h postinfection (hpi) for the following studies.

African green monkey kidney (Vero) cells for viral plaque assays were obtained from the Institute of Medical Biology, Kunming, China, and maintained in minimum essential medium (MEM) with 10% newborn bovine serum (NBS, Gibco, USA), 2 mM L-glutamine, and 1% penicillin/streptomycin at 37 °C in a humidified cell culture incubator with 5% CO_2_.

### Plasmids and transfection

The plasmids pcDNA3.1-EGFP-LC3 and pcDNA3.1-EGFP-mCherry-LC3 used in the current study were previously stored in our lab. Cells were seeded on chamber slides that were pre-placed in 6-well plates at a concentration of 2 × 10^5^ cells/well. When they were approximately 80% confluent, cells were transfected with these plasmids using FuGENE^®^ HD Transfection Reagent (Promega, USA) according to the manufacturer’s instructions. After 48 h of transfection, the efficiency of transfection was determined based on fluorescence with a fluorescence microscope, and the cells then were treated with EV71, EV71+3-MA (the concentration of 3-MA treatment was 10 mM), CA16, CA16+3-MA, rapamycin or NH_4_Cl for 24 h.

### Immunofluorescence (IF) staining

Autophagosome formation in 16HBE cells with different treatments was examined by IF staining. At the indicated times, cells plated on chamber slides were washed twice with phosphate-buffered saline (PBS) and fixed with 4% paraformaldehyde (PFA) (Solarbio, China) for 30 min at room temperature and were then rinsed with PBS. After permeabilizing with 1% Triton X-100 in PBS for 15 min and blocking with 5% bovine serum albumin (BSA) for 1 h at room temperature, cells were incubated overnight at 4 °C with primary antibodies against EV71/CA16-VP1 (1:1000; Millipore, USA), LC3 (1:1000; Cell Signaling Technology, USA), TLR7 (1:1000; Abcam, USA) or M6PR (1:1000; Abcam, USA) diluted in a blocking solution. The next day, the cells were washed three times with PBS, and Alexa Fluor 647–conjugated donkey anti-mouse IgG (diluted 1:1000; Millipore, USA) and Alexa Fluor 488–conjugated donkey anti-rabbit IgG (diluted 1:1000; Biolegend, USA) were added to the cells, which were then incubated at 37 °C for 1 h in the dark and washed with PBS. Finally, the nuclei were counterstained with 4’, 6-diamidino-2-phenylindole (DAPI, 1:4000, Beyotime, China), and the slides were mounted with antifade reagent (Solarbio, China). The images were visualized and captured with a laser-scanning confocal microscope (Leica, Germany) using the appropriate filters.

### Cell proliferation assay

A Cell Counting Kit-8 (CCK-8) (Dojindo Molecular Technologies, Japan) was used to assess the effects of EV71, CA16, 3-MA and rapamycin on the viability of 16HBE cells. Briefly, 16HBE cells were resuspended at a density of 1 × 10^5^ cells/ml, added to a 96-well plate (100 μl/well) in triplicate, incubated at 37 °C in a 5% CO_2_ incubator overnight, and treated as described earlier. After 0 h, 6 h, 12 h and 24 h of treatment, 10 μl of CCK-8 reagent was added to each well, and the cells were further incubated for an additional 4 h. The absorbance values were examined using a microplate reader (Bio-Rad, USA) at a wavelength of 450 nm. The cell survival rate was calculated according to the following equation: Cell survival rate (%) = [(absorbance value of treatment group − the absorbance of the blank medium)/(absorbance value of the mock-treated sample − the absorbance of the blank medium)] × 100.

### RNA isolation and quantitative reverse transcription polymerase chain reaction (qRT-PCR)

All samples were harvested at the designated time, and total RNA was then extracted using TRIzol Reagent (TIANGEN, China) according to the manufacturer’s guidelines. The quality and concentrations of total RNA were determined using a BioPhotometer, and the integrity of the total RNA was assessed by agarose-formaldehyde gel electrophoresis. qRT-PCR was carried out using a 7500 Fast Real-time PCR system (Applied Biosystems, USA) with a One Step SYBR^®^ PrimeScript™ RT-PCR Kit (Takara, Japan) under the following conditions: 1 cycle at 42 °C for 5 min and 1 cycle at 95 °C for 10 s, followed by a two-step procedure consisting of 5 s at 95 °C and 34 s at 60 °C for 40 cycles (with data collection at the end of the 60 °C step at each cycle), and dissociation at 95 °C for 60 s, 55 °C for 30 s and 95 °C for 30 s. The primers used for qRT-PCR are listed in Table S1. The fold change in the mRNA expression levels of these genes was calculated using the 2^-ΔΔCt^ method of relative quantification using GAPDH as the endogenous reference gene. All reactions, including no-template controls, were carried out in triplicate.

### Measurement of the IFN-α and IFN-β levels in 16HBE cells infected with EV71 and CA16

All collected samples were centrifuged at 1500 rpm for 10 min at 4 °C, and the cell-free supernatant was immediately stored at -80 °C until the experiment was performed. The concentrations of IFN-α and IFN-β were measured by “sandwich” enzyme-linked immunosorbent assay (ELISA) using commercially available kits, namely, human IFN-α ELISA (R&D, UK) and human IFN-β ELISA (R&D, UK), according to the manufacturer’s instructions. The results were normalized to the amount of conditioned media and expressed as pg/ml.

### Western blotting (WB)

Whole-cell protein lysates were solubilized in 100-200 μl of radioimmunoprecipitation assay (RIPA) lysis buffer (Beyotime, China) supplemented with a 1% protease inhibitor cocktail (Beyotime, China) for 20 min on ice. The protein concentration was determined using a BCA protein assay kit (Beyotime, China). The proteins (30 μg) were denatured and separated on an 8%-15% discontinuous SDS-polyacrylamide gel (SDS-PAGE) by electrophoresis and subsequently electrophoretically transferred to a polyvinylidene difluoride (PVDF) membrane. After blocking with 5% non-fat dry milk in Tris-buffered saline with 0.1% Tween-20 (TBST) for 2 h at room temperature, the PVDF membrane was incubated overnight at 4 °C with the appropriate primary antibody for TLR7 (1:100; Novusbio, USA), MyD88 (1:200; Boster, China), IRF7 (1:1000; Abcam, USA), beclin 1 (1:200; Boster, China), SQSTM1 (1:200; Boster, China), EV71/CA16-VP1 (1:1000; Millipore, USA) or GAPDH (as a loading control, 1:10000; Abmart, China). Then, the membrane was extensively washed three times with TBST prior to incubation for 1 h at room temperature with the corresponding secondary antibody (Abmart, USA) at a dilution of 1:12,000. Finally, the membrane was again extensively washed three times with TBST and then visualized using enhanced chemiluminescence reagents (Beyotime, China) and X-ray films (Kodak, Japan).

### Plaque assay

Viral titers were measured using a standard plaque assay on Vero cells. Monolayer cultures of Vero cells grown in a six-well plate were infected with tenfold serial dilutions of the thawed samples (1 ml per well) for 3 h to allow virus attachment. After removing the viral inoculum, the wells were gently washed with PBS, covered with fresh DMEM containing 2% FBS with 0.6% low-melting-point agarose, and kept in a 37 °C CO_2_ incubator for 48 h until plaques were visible. Next, the cells were fixed with 2 ml of 4% PFA for 30 min, followed by the removal of agarose plugs and staining with a crystal violet staining solution for 15 minutes at room temperature. The visible plaques were counted, and the number of plaque-forming units (pfu/ml) was calculated using the virus titer formula: virus titer = the number of plaques × (1 ml) × dilution factor. Three independent replicates of each sample were tested in this assay.

### Statistical analysis

All statistical calculations were carried out using SPSS 18.0 software (IBM SPSS, USA). The values are expressed as the mean ± the standard error of the mean (SEM) from three independent experiments. A two-tailed Student’s *t-*test and one-way analysis of variance (ANOVA) were applied to analyze data between two and more than two groups, respectively. The results were considered statistically significant when the value of *P* was less than 0.05.

## Results

### Induction of different types of autophagy in 16HBE cells by EV71 and CA16 infection

**Fig. 1:**
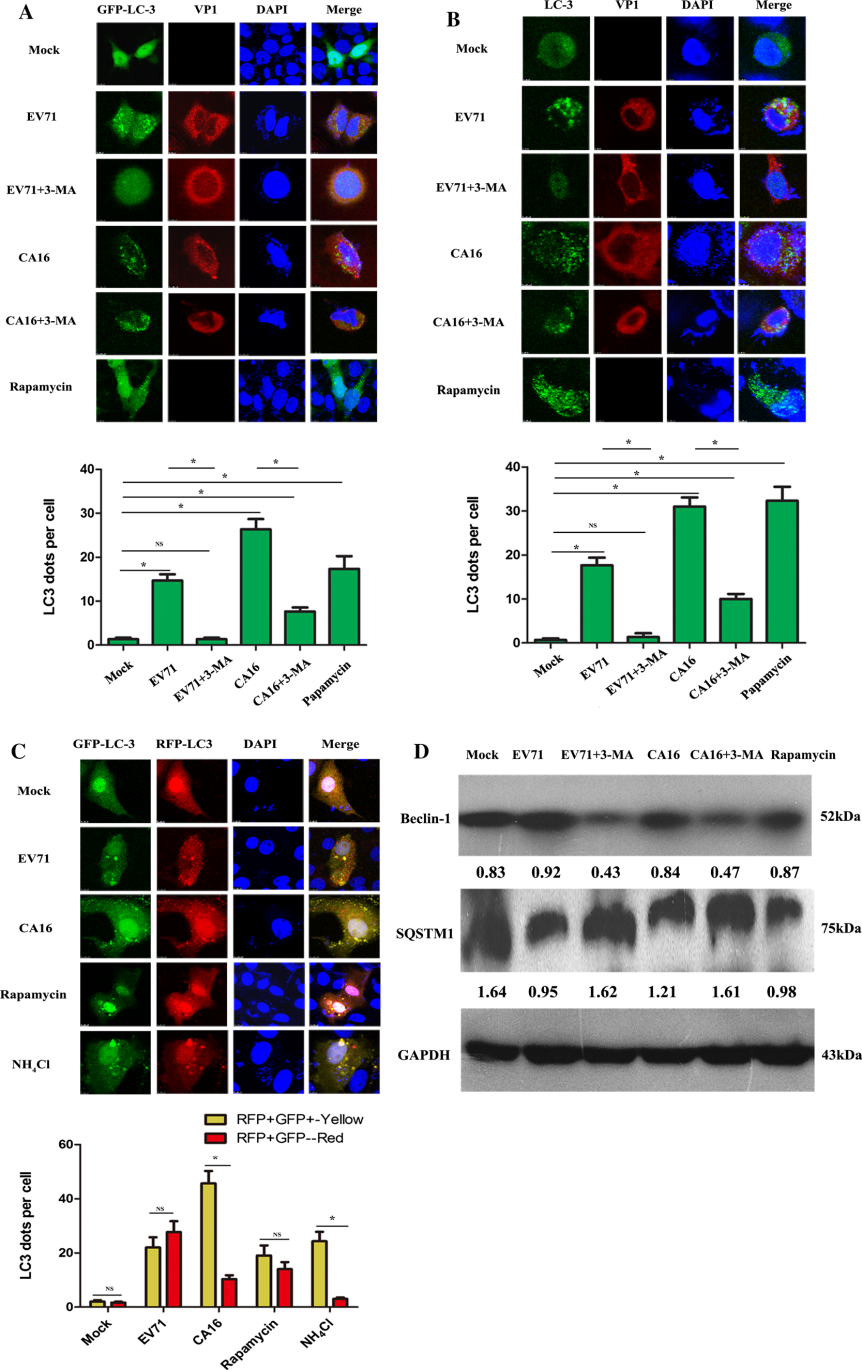
EV71 and CA16 infections trigger autophagy in 16HBE cells. **A.** GFP-LC3 dots and viral protein expression visualized by confocal microscopy. Quantification of exogenous LC3 puncta was performed using ImageJ software, using at least 20 cells in each sample. **B**. Endogenous LC3 dots and viral protein expression visualized by confocal microscopy. Quantification of exogenous LC3 puncta was performed using Image J software with at least 20 cells in each sample. **C**. Autophagy flux examined by transfecting 16HBE cells with the plasmid a pcDNA3.1-EGFP-mCherry-LC3, followed by different treatments. Yellow dots represent autophagosomes, and red dots indicate autolysosomes. The average number of yellow or red dots was calculated by Image J software, with at least 20 cells in each group. **D.** Autophagy-related proteins, relative to GAPDH control, were analyzed by WB. The band intensity values are shown under each band. *, *P* < 0.05; NS, not significant

To determine whether EV71 and CA16 can induce autophagy in 16HBE cells, exogenous and endogenous LC3 were observed using a laser-scanning confocal microscope. The transfection efficiency of exogenous LC3, including GFP-LC3 and EGFP-mCherry-LC3 plasmids, was determined using a fluorescence microscope, and the results are shown in Fig. S1. As shown in Fig. 1A, rapamycin-treated cells, which were used as a positive control, exhibited apparent GFP-LC3 puncta that corresponded to autophagosomes, and these were not present in mock-treated cells. The infected cells also displayed a greater number of GFP-LC3 puncta; when treated with 3-MA for 3 h prior to EV71 or CA16 infection, GFP-LC3 puncta completely disappeared in the 3-MA-treated EV71-infected cells and were reduced in number in the 3-MA-treated CA16-infected cells. Subsequently, to eliminate the influence of exogenous factors on pcDNA3.1-EGFP-LC3 plasmid transfection, we examined the location of endogenous LC3 in 16HBE cells subjected to different treatments and found a similar pattern, as shown in Fig. 1B. These results suggested that EV71 and CA16 could induce autophagy in 16HBE cells.

To evaluate the differences between EV71 and CA16 in their ability to induce autophagy, we transfected 16HBE cells with a pcDNA3.1-EGFP-mCherry-LC3 plasmid, which labels autophagosomes with both red and green fluorescent puncta. Autolysosomes are only labeled with red due to the degradation/quenching of acid-labile GFP in lysosomes. As shown in Fig. 1C, the numbers of yellow (autophagosomes) and red (autolysosomes) LC3 puncta per cell in merged images were significantly increased in 16HBE cells infected with EV71, and there were no differences between the numbers of yellow and red LC3 puncta, which indicated that approximately one-half of autophagosomes were diffused into autolysosomes. This phenomenon was similarly observed in rapamycin-treated cells, which represented complete autophagy. However, the number of yellow LC3 puncta was markedly higher than the number of red LC3 puncta in 16HBE cells following CA16 infection, which indicated that only a small proportion of autophagosomes diffused into autolysosomes. This phenomenon was similarly observed in NH_4_Cl-treated cells, which represented an incomplete autophagy process. Hence, these findings implied that EV71 infection might trigger complete autophagy in 16HBE cells, but CA16 infection might trigger incomplete autophagy in 16HBE cells.

Finally, we investigated the effects of EV71 and CA16 on autophagic flux by assessing the protein levels of SQSTM1 (also known as P62) and beclin 1. EV71 and CA16 infections concomitantly reduced the SQSTM1 protein levels with an increase in the beclin 1 protein levels compared to the mock-treated cells. However, after pre-treatment with 3-MA, there were reduced SQSTM1 levels accompanied by a decrease in the beclin 1 protein levels compared to the mock-, EV71-, and CA16-infected cells (Fig. 1D). Taken together, these data confirm that infection with EV71 or CA16 leads to autophagy in 16HBE cells.

### Effects of autophagy induced by EV71 and CA16 on the 16HBE cell survival rate

**Fig. 2:**
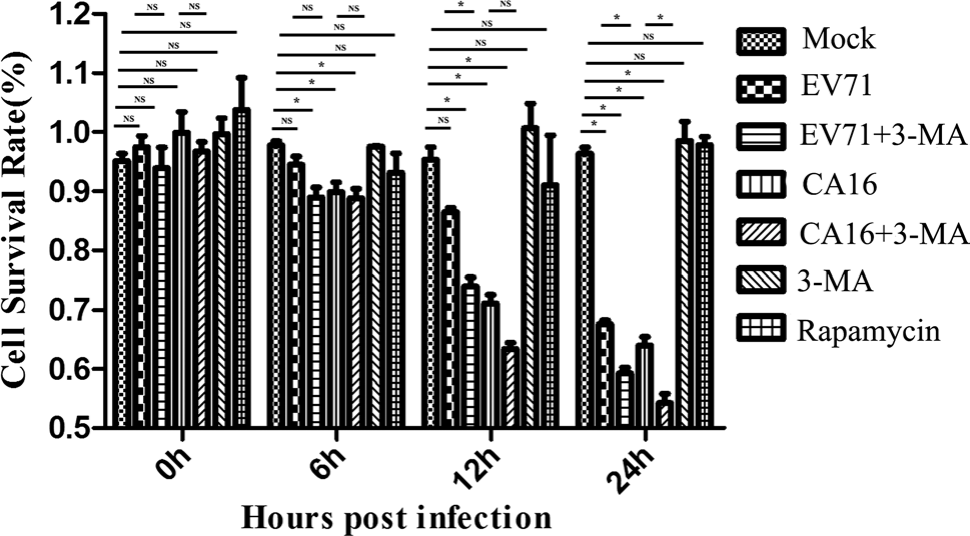
Autophagy inhibitor 3-MA promotes survival of EV71 and CA16 in 16HBE cells, as determined by CCK-8 assay. *, *P* < 0.05; NS not significant

The results of the CCK-8 assay revealed that there were no changes in the cell survival rate in the rapamycin group at different time points. Moreover, compared to mock-treated cells, the cell survival rate was gradually decreased over time in EV71-, EV71+3-MA-, CA16- and CA16+3-MA-treated cells. In addition, at 12 h and 24 h postinfection, the cell survival rate in the EV71+3-MA-treated cells was much lower than that in EV71-infected cells without 3-MA. At 24 h postinfection, the cell survival rate in the CA16+3-MA-treated cells was similarly lower than that of CA16-infeced cells without 3-MA (Fig. 2). Thus, these data demonstrated that autophagy induced by EV71 and CA16 promoted cell survival, but the cell survival rate dramatically declined when autophagy was inhibited.

### Increase in viral replication due to EV71- and CA16- induced autophagy

**Fig. 3:**
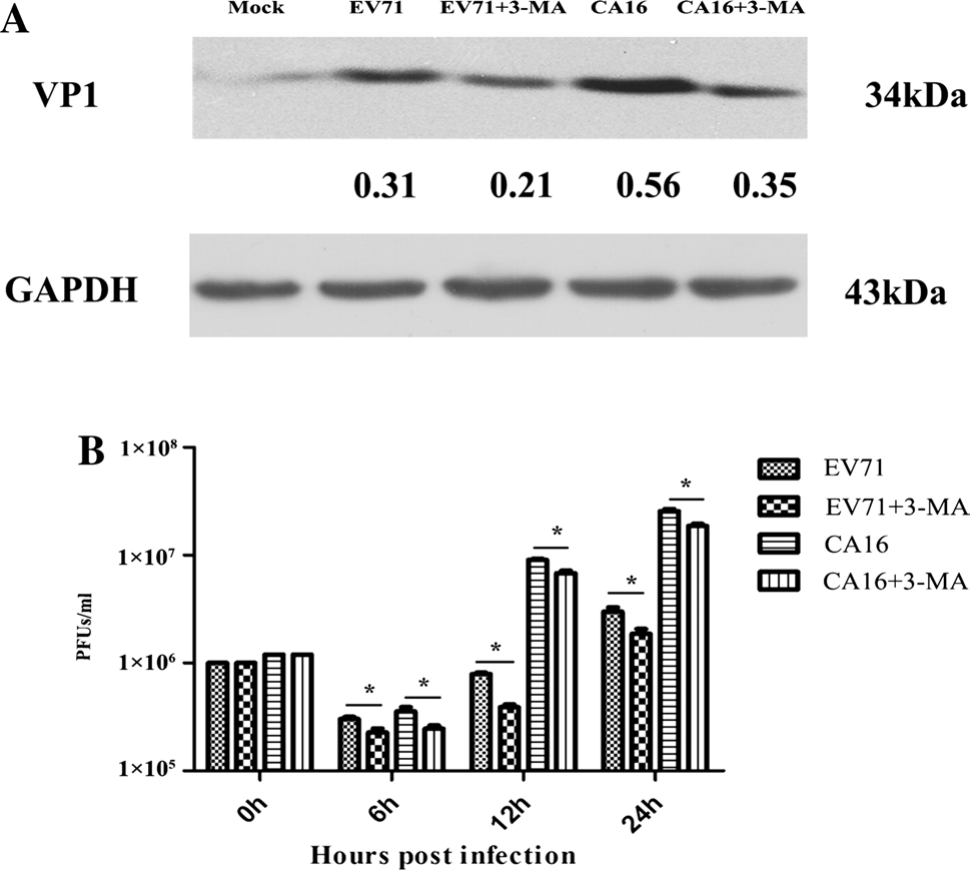
Autophagy facilitates the replication of EV71 and CA16 in 16HBE cells. **A.** VP1 protein of EV71 and CA16 measured by WB. GAPDH was used as an internal control. The band intensity values are shown under each band. **B.** Viral titers tested by plaque assay at various times

VP1 expression detected by WB and viral titers examined by plaque assays demonstrated that, after pre-treatment with 3-MA, VP1 expression and viral titers were clearly downregulated compared to untreated cells (Fig. 3), suggesting that suppression of autophagy might alleviate EV71 and CA16 infections.

### Changes in expression of IFN-related molecules in 16HBE cells due to EV71- and CA16-induced autophagy

**Fig. 4:**
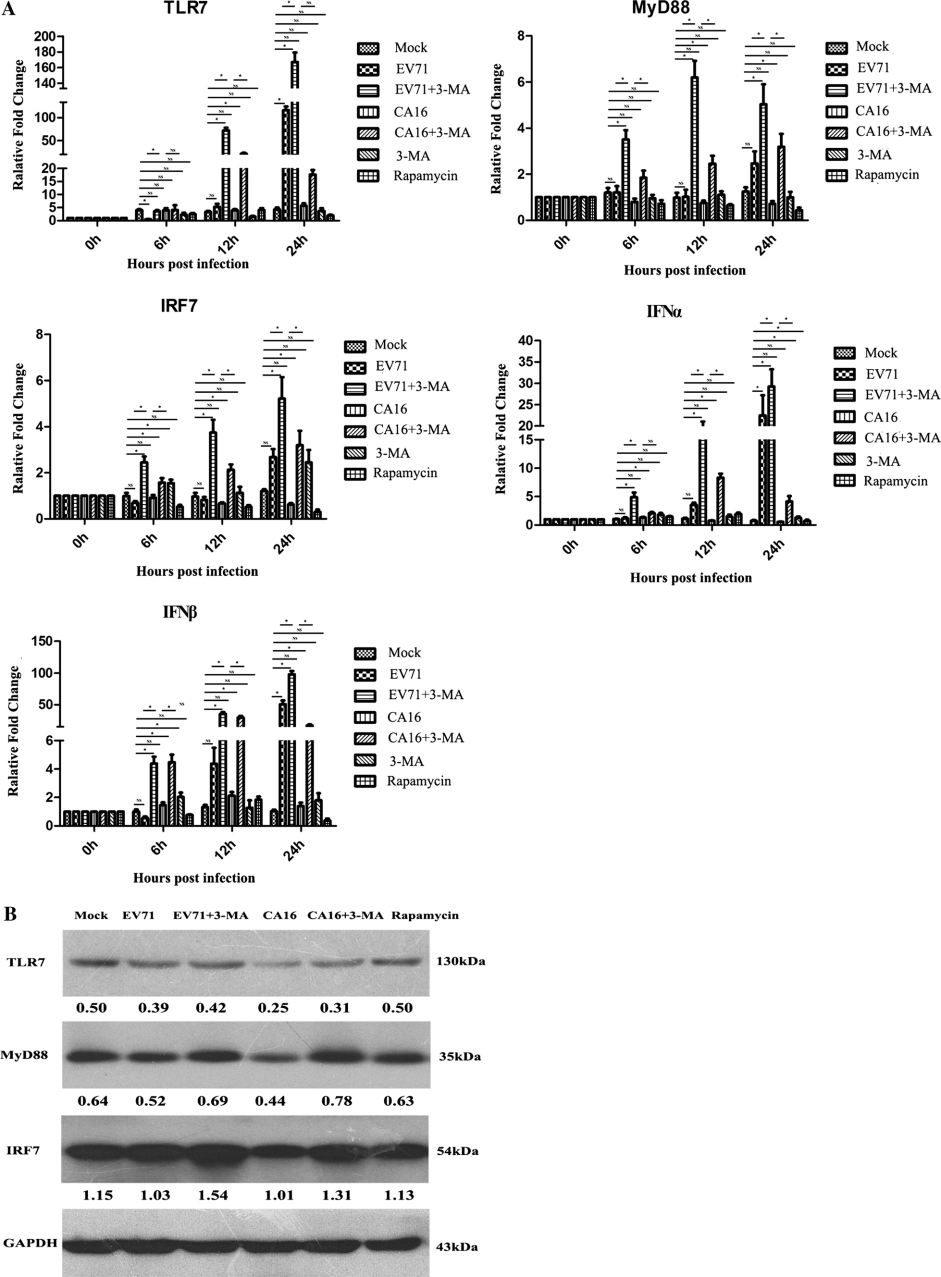
Autophagy suppresses the TLR7-dependent IFN-I production pathway in EV71 and CA16 infections. **A.** TLR7-dependent IFN-I mRNA expression examined by qRT-PCR (normalized to GAPDH) in 16HBE cells with different treatments at 0 h, 6 h, 12 h and 24 h. ^*^, *P* < 0.05; NS, not significant **B.** TLR7-dependent IFN-I protein expression detected by WB (normalized to GAPDH) in 16HBE cells with different treatments at 0 h, 6 h, 12 h and 24 h. Band intensity values are shown under each band

To explore the effect of autophagy caused by EV71 and CA16 infection on the production of IFN-related proteins in 16HBE cells, the levels of mRNA encoding these proteins were measured by qRT-PCR. The levels of IFN-related mRNAs were altered after infection with EV71 or CA16. However, expression of TLR7, MyD88, IRF7 and IFN-α/β mRNA was significantly higher in EV71- or CA16-infected cells treated with 3-MA than in untreated infected cells. This effect was stronger in EV71-infected cells than in CA16-infected cells (Fig. 4A). These results suggest that autophagy resulting from EV71 and CA16 infection might negatively regulate TLR7, MyD88, IRF7 and IFN-α/β expression, ultimately resulting in immune evasion by EV71 and CA16, and that the antiviral response is enhanced when autophagy is suppressed.

To further examine the effects of autophagy caused by EV71 and CA16 infection on the expression of IFN-related proteins in 16HBE cells, the levels of these proteins were assayed by WB analysis and ELISA. WB analysis indicated that the expression of TLR7, MyD88 and IRF7 was lower in EV71- and CA16-infected cells, but higher in infected cells treated with 3-MA than in mock-infected and rapamycin-treated cells (Fig. 4B). IFN-α was not detected in mock-infected or rapamycin-treated cells, but it was expressed at higher levels in infected cells treated with 3-MA than in IFN-α in EV71+3-MA and CA16+3-MA groups were apparently higher than those in EV71- or CA16-infected cells without 3-MA treatment (Fig. S2A). IFN-β was not detected under any of the experimental conditions (Fig. S2B), probably due to lower expression levels. The changes in protein expression levels corresponded to the changes in mRNA expression levels, supporting the qRT-PCR results.

### Autophagy resulting from EV71 and CA16 infection in 16HBE cells probably suppresses TLR7 by reducing endosome formation

**Fig. 5:**
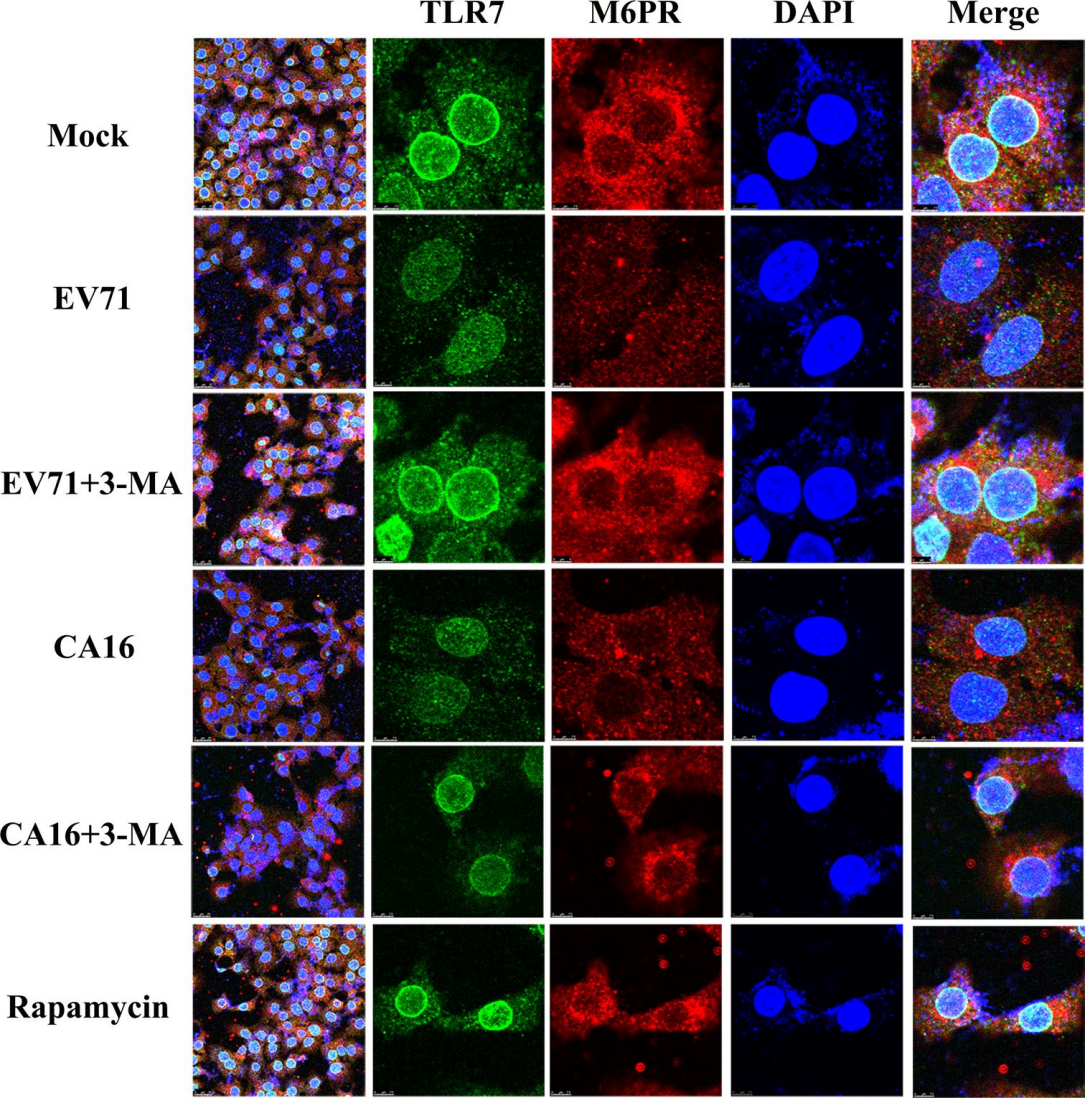
Autophagy inhibits the TLR7 signaling pathway by disrupting endosome formation. Cells were fixed and permeabilized and then stained with antibodies to TLR7 (green), M6PR (red), or DAPI (blue) (color figure online)

To address how EV71- and CA16-induced autophagy in 16HBE cells leads to the inhibition of TLR7, which is primarily located in endosomes, M6PR (an endosome marker) was detected by IF staining. As shown in Fig. 5, M6PR and TLR7 expression was markedly decreased in 16HBE cells after EV71 or CA16 infection, but M6PR expression recovered significantly, concomitant with an increase in TLR7, after treatment with 3-MA. Therefore, a decrease in endosome formation might be an important reason for the decrease in TLR7 expression in 16HBE cells infected with EV71 or CA16.

## Discussion

Autophagy is an evolutionarily conserved catabolic process that is primarily responsible for the maintenance of cellular homeostasis and cell survival by clearing intracytoplasmic components and dysregulated organelles in the lysosome under circumstances such as hypoxia, nutrient deprivation, oxidative stress, and exposure to xenobiotics [17]. Many preclinical studies have demonstrated that autophagy is closely associated with cancers, cardiovascular disease, neurodegenerative disorders, aging, inflammatory bowel disorders, and infectious diseases [18]. Therefore, in recent years, many researchers have invested a significant amount of effort in searching for autophagy regulators as a pathogenic mechanism and drug target for these human diseases [19]. An increasing body of evidence suggests that autophagy and/or autophagy execution genes are a “double-edged sword” for viral infectious diseases, as they can either promote or suppress viral replication in host cells [20]. For instance, autophagy exerts a direct antiviral effect against the mammalian viral pathogen vesicular stomatitis virus (VSV) in the model organism *Drosophila* [21]. However, influenza A virus infection *in vitro* induces autophagy and autophagic flux, which function in a pro-viral capacity by increasing viral replication [22]. The autophagic machinery is triggered in infections with enteroviruses, including EV71 and CA16, but to date, the exact role of autophagy is still unclear [13, 14]. In the present study, we demonstrate that EV71 infection elicits complete autophagy, whereas CA16 infection induces incomplete autophagy, based on observations obtained by IF staining. Moreover, viral replication of EV71 and CA16 was observed to be significantly increased by autophagy. These results are consistent with earlier studies by Lee et al. and Shi et al., which suggested that autophagy might have a pro-viral role in the life cycles and pathogenesis of EV71 and CA16 infections [13, 14]. To confirm this conclusion, cells were pre-treated with the autophagy-related inhibitor 3-MA before infection with EV71 or CA16. Inhibition of autophagy clearly reduced viral replication, and cell survival rates were clearly decreased when autophagy was suppressed. These findings suggest that autophagy is a direct factor in EV71 and CA16 replication.

There is evidence of a vital link between autophagy and host pattern recognition receptor (PRR)-mediated innate immunity, especially TLRs [23]. For example, TLR7, which is present in cellular endosomal compartments, can recognize single-stranded (ss)-RNA from exogenous viruses and trigger the activation of MyD88-IRF7 signaling to further induce the formation of autophagosomes, which can directly combat pathogen invasion [24, 25]. This suggests that activation of TLRs facilitates pathogen elimination by induction of autophagy. In contrast, the autophagic machinery can also be activated directly by exogenous pathogens causing it to deliver pathogen-associated molecular patterns (PAMPs) to TLRs as a defense against the invading pathogens [26]. In the case of vesicular stomatitis virus (VSV), pDCs recognize replicating virus in the cytosol, and subsequently, these cytosolic replication intermediates were wrapped in an autophagosome, which allows them to gain entry to the endosomal compartment where TLR7 resides. Eventually, autophagy mediates the delivery of the cytosolic PAMP to lysosomes to activate TLR7 signaling [27]. These observations implied that autophagy functions as a direct effector for protection against pathogens, as well as a modulator of pathogen recognition and downstream TLR signaling in innate immune responses [28]. In this work, to explore how autophagy enhances EV71 and CA16 replication, we focused on TLR7. Our data show that the gene and protein expression levels of TLR7 signaling-related molecules in EV71- and CA16-infected 16HBE cells were markedly lower than in EV71- and CA16-infected 16HBE cells that were pretreated with 3-MA. These results suggest that autophagy inhibits the TLR7-dependent IFN-I production pathway in EV71 and CA16 infections. Furthermore, TLR7 and an endosome marker, M6PR, were notably present when autophagy was suppressed, implying that autophagy might inhibit the TLR7 signaling pathway by degrading the endosome. Thus, it is speculated that lower concentrations of secreted IFN-I, a downstream product of the TLR7 signaling pathway, enables EV71 and CA16 to escape the innate immune response and facilitates replication. However, it has been reported that ligands of TLR3 and TLR7 cause autophagosome formation in murine macrophages via myeloid differentiation primary response gene 88 (MyD88), which eventually leads to the destruction of intracellular microbes [29]. This is a further demonstration of the “double-edged sword” of autophagy in pathogen invasion and the close relationship between autophagy and the TLR signaling pathway.

In conclusion, this study demonstrates that different degrees of autophagy induced by EV71 and CA16 infection hinders IFN-I production by promoting endosomal degradation and inhibiting the TLR7 signaling pathway, ultimately leading to successful replication of EV71 and CA16 in host cells. This suggests that autophagy might be a novel and effective therapeutic target against EV71 and CA16 infections.

## Electronic supplementary material

Below is the link to the electronic supplementary material.

**Fig. S1** The transfection efficiency of LC3, including GFP-LC3 and EGFP-mCherry-LC3 plasmids, calculated from fluorescence microscopy data (TIFF 5756 kb)

**Fig. S2** IFN-α/β protein levels determined by ELISA (TIFF 3010 kb)

Supplementary material 3 (DOCX 13 kb)
