# Supplementary figures and images for: Suppression of the toll-like receptor 7-dependent type I interferon production pathway by autophagy resulting from enterovirus 71 and coxsackievirus A16 infections facilitates their replication
Source: Arch Virol. 2017 Oct 19;163(1):135–44. doi: 10.1007/s00705-017-3592-x (PMC5756282; doi:10.1007/s00705-017-3592-x)

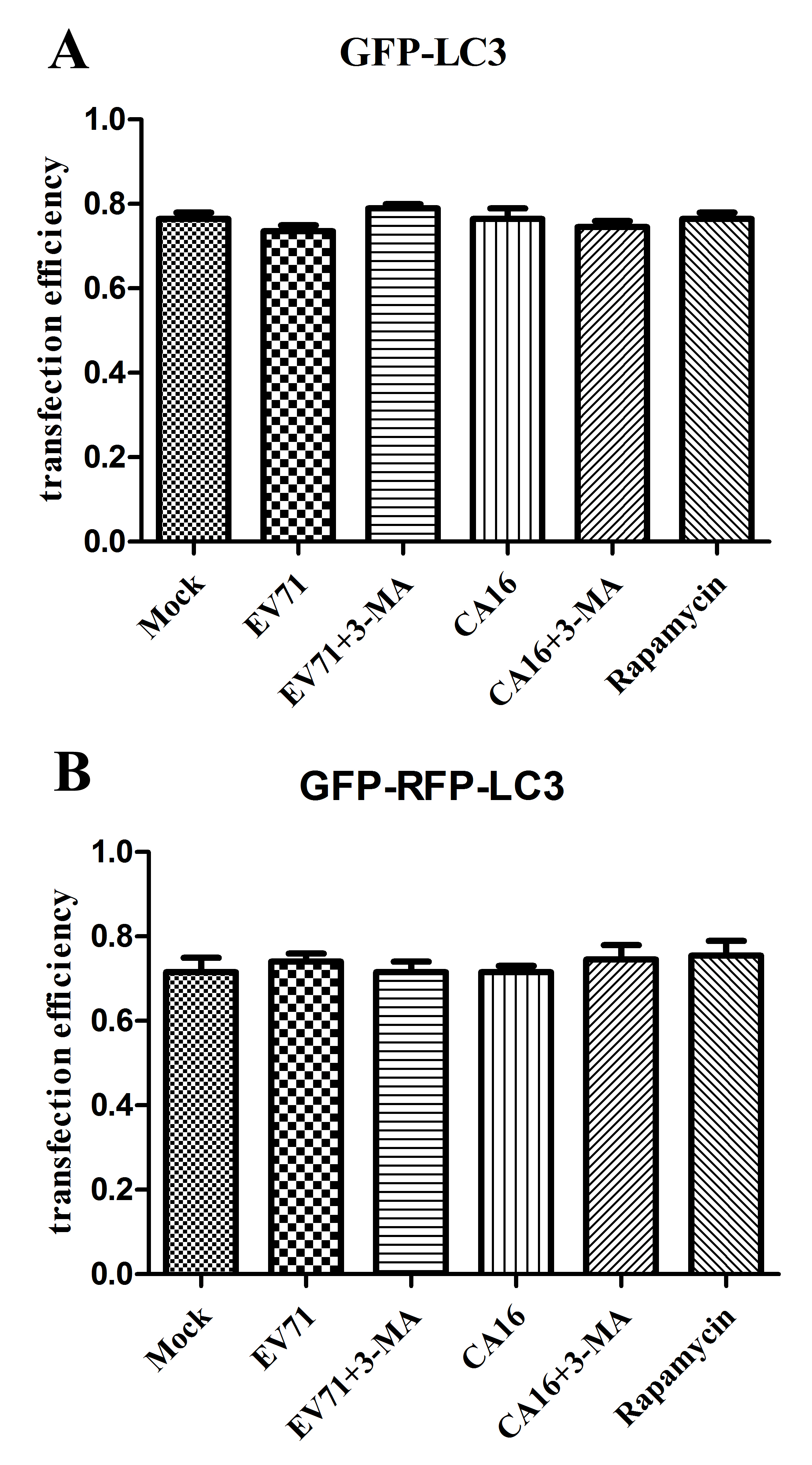

Supplement: Supplementary file 1 — Fig. S1 The transfection efficiency of LC3, including GFP-LC3 and EGFP-mCherry-LC3 plasmids, calculated from fluorescence microscopy data (TIFF 5756 kb) [file 705_2017_3592_MOESM1_ESM.tif]

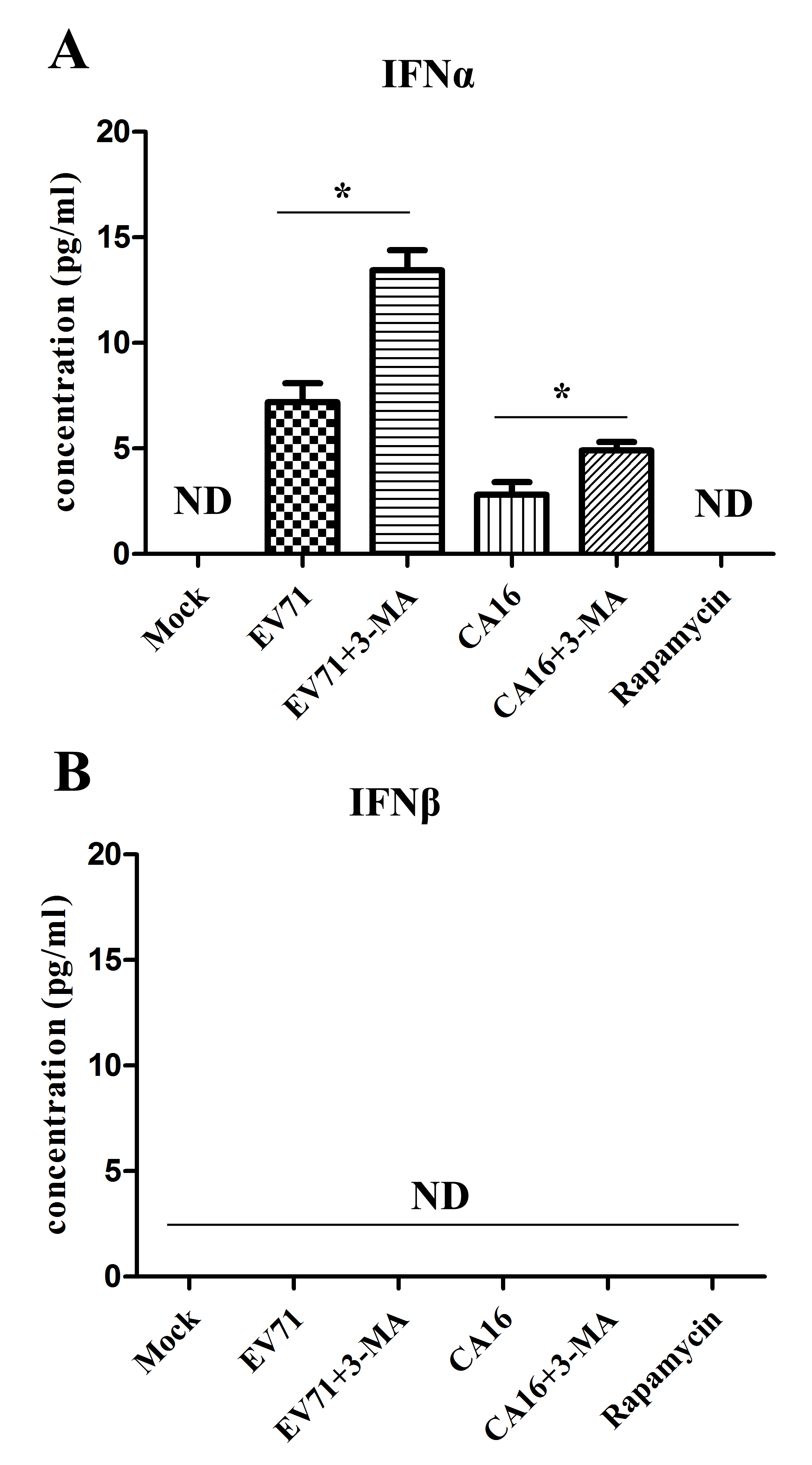

Supplement: Supplementary file 2 — Fig. S2 IFN-α/β protein levels determined by ELISA (TIFF 3010 kb) [file 705_2017_3592_MOESM2_ESM.tif]
