# Supplementary material for: Suppression of the toll-like receptor 7-dependent type I interferon production pathway by autophagy resulting from enterovirus 71 and coxsackievirus A16 infections facilitates their replication
Source: Arch Virol. 2017 Oct 19;163(1):135–44. doi: 10.1007/s00705-017-3592-x (PMC5756282; doi:10.1007/s00705-017-3592-x)
Supplement: Supplementary file 3 — Supplementary material 3 (DOCX 13 kb) [file 705_2017_3592_MOESM3_ESM.docx]

**Table S1. Sequences of primers used for qRT-PCR assays.**

| **Genes** | **Primer sequences** |
| --- | --- |
| TLR7 | Forward primer: 5’- CCTTGTGCGCCGTGTAAAAA -3’  Reverse primer: 5’- GGGCACATGCTGAAGAGAGT -3’ |
| MyD88 | Forward primer: 5’- TCATCACTGTCTGCGACTACACC -3’  Reverse primer: 5’- GAGCACAGATTCCTCCTACAACGA -3’ |
| IRF7 | Forward primer: 5’- CCCCAGCAGGTAGCATTCCC -3’  Reverse primer: 5’- CGAAGCTCCAGGTGCAACCC -3’ |
| IFNα | Forward primer: 5’- ACCCCTGCTATAACTATGACC -3’  Reverse primer: 5’- CTAACCACAGTGTAAAGGTGC -3’ |
| IFNβ | Forward primer: 5’- TTGCTCTCCTGTTGTGCTT -3’  Reverse primer: 5’- GCTGCTTCTTTGTAGGAATCCA -3’ |
| GAPDH | Forward primer: 5’- AGAAGGCTGGGGCTCATTTG -3’  Reverse primer: 5’- AGGGGCCATCCACAGTCTTC -3’ |
